# Ectopic adrenal tissue in the mesosalpinx: a rare incidental finding in an adult woman

**DOI:** 10.1093/jscr/rjag071

**Published:** 2026-03-07

**Authors:** Behrooz Shokouhi, Ali Kazemiathar

**Affiliations:** Department of Pathology, Alzahra Hospital, Tabriz University of Medical Sciences, Baghe Shomal Street, Tabriz, East Azerbaijan Province, 5138663134, Iran; Department of Pathology, Alzahra Hospital, Tabriz University of Medical Sciences, Baghe Shomal Street, Tabriz, East Azerbaijan Province, 5138663134, Iran

**Keywords:** ectopic adrenal tissue, adrenal rest, mesosalpinx, fallopian tube, histopathology

## Abstract

Ectopic adrenal tissue is most often identified incidentally on histopathological examination. It is reported mainly in male children and is usually located within the abdominopelvic cavity. Although adrenal cortical rests have been described in the female genital tract, involvement of the mesosalpinx (paratubal region) is exceptionally rare. We report a microscopic adrenal cortical rest in the left mesosalpinx of a 47-year-old woman who underwent total abdominal hysterectomy with bilateral salpingo-oophorectomy for abnormal uterine bleeding and evaluation of bilateral complex ovarian cysts. The ectopic rest formed a well-circumscribed 3-mm nodule composed of bland polygonal cells with clear to eosinophilic cytoplasm, consistent with adrenal cortex. This case underscores the value of careful gross sampling and microscopic review in detecting uncommon developmental anomalies that are clinically silent.

## Introduction

The adrenal gland is an encapsulated endocrine organ located superior to the kidneys and composed of cortex and medulla [1]. Ectopic adrenal tissue (adrenal rest) represents displaced adrenal tissue outside its normal anatomic site. It is commonly detected in the inguinoscrotal region of infants and children, where its prevalence has been reported to be high, but it becomes uncommon in adults because the rests typically regress after birth [2, 3]. In adults, ectopic adrenal tissue has been described along the urogenital tract, reflecting the close embryologic relationship between the adrenal primordium and the developing gonads; however, localization to the mesosalpinx is rare [4, 5]. Here we describe an incidental adrenal cortical rest identified in the mesosalpinx of an adult woman undergoing hysterectomy with bilateral salpingo-oophorectomy.

## Case report

A 47-year-old woman presented with an 8-year history of irregular vaginal bleeding refractory to medical management. Pelvic ultrasonography and magnetic resonance imaging demonstrated a myomatous uterus with multiple leiomyomas and bilateral complex ovarian cysts classified as Ovarian-Adnexal Reporting and Data System (O-RADS) category 3–4. Given persistent symptoms and the low-to-intermediate malignancy risk suggested by imaging, total abdominal hysterectomy with bilateral salpingo-oophorectomy was performed.

Gross examination showed a markedly myomatous uterus with multiple well-circumscribed intramural leiomyomas. The endometrium was thin and irregular with a small polypoid lesion. Both ovaries were enlarged with multiloculated cystic change, and the fallopian tubes were grossly unremarkable.

**Figure 1:**
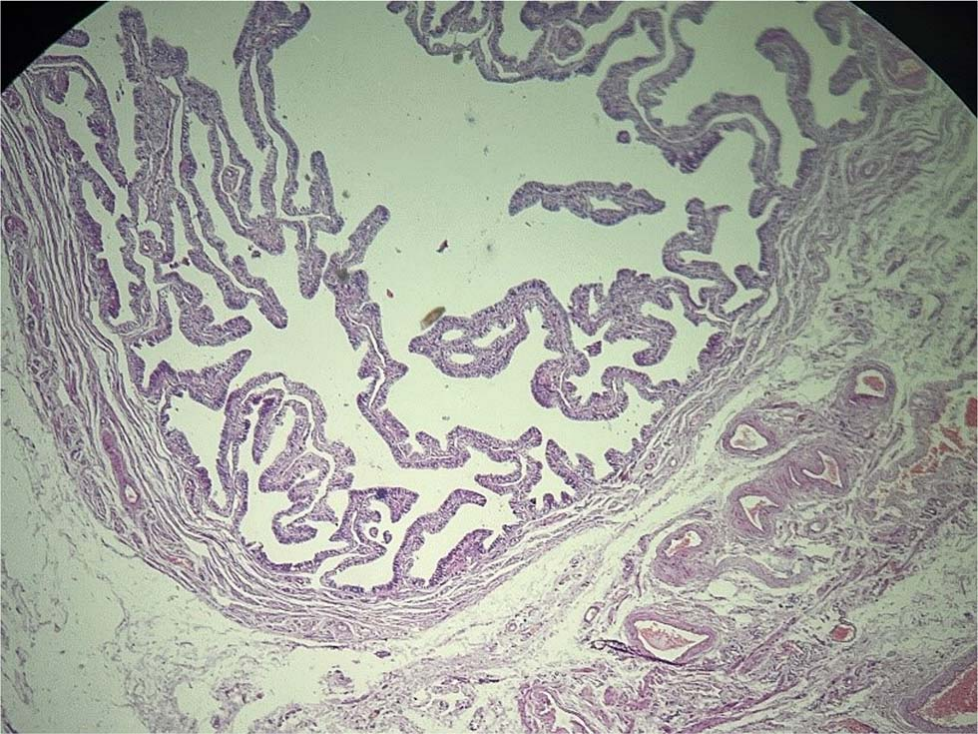
Left fallopian tube and adjacent mesosalpinx without significant histopathologic abnormality. Hematoxylin and eosin (H&E) stain, ×40.

**Figure 2:**
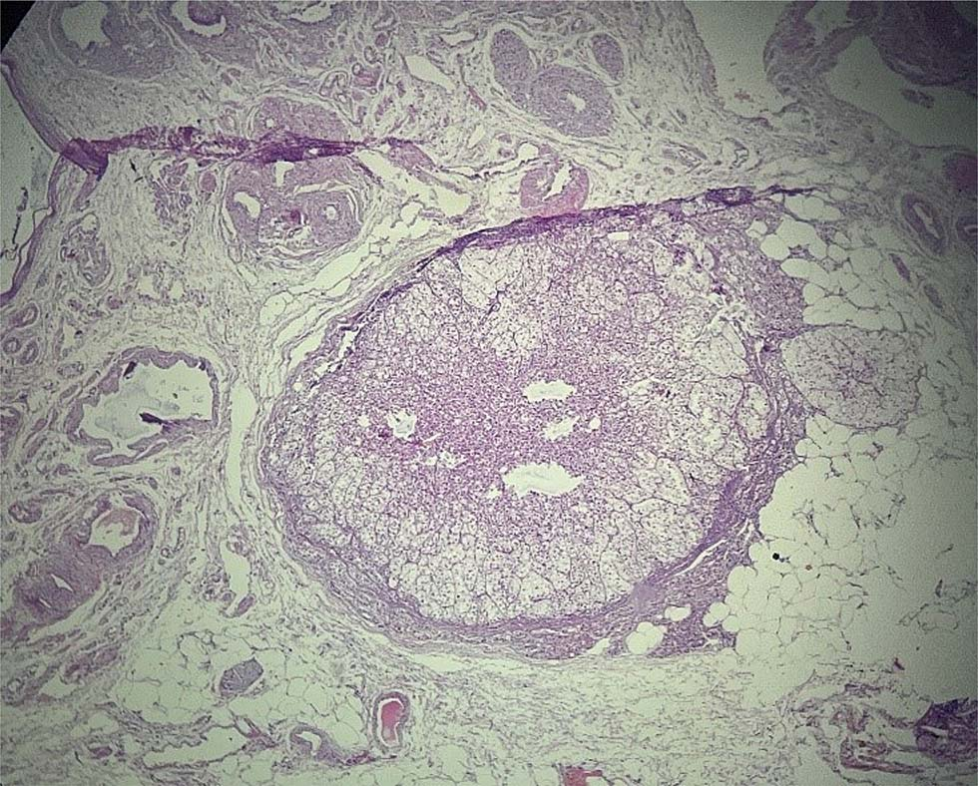
Ectopic adrenal cortical tissue in the mesosalpinx, forming a well-circumscribed nodule without invasive features. H&E stain, ×40.

**Figure 3:**
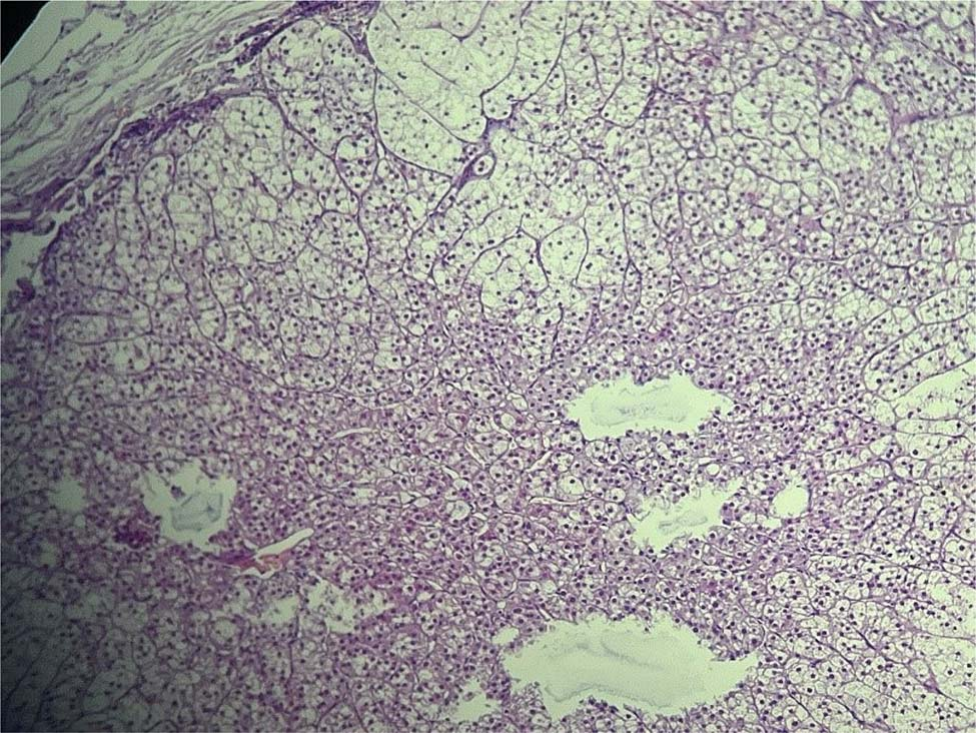
High-power view of ectopic adrenal cortex composed of polygonal cells with finely vacuolated, lipid-rich cytoplasm, and round to oval nuclei, arranged in nested/trabecular patterns resembling zona fasciculata. No medullary tissue is present. H&E stain, ×100.

Microscopic evaluation (hematoxylin and eosin) demonstrated an endometrial polyp without atypia, multiple leiomyomas, and bilateral benign serous cystadenomas. Sections from the left fallopian tube and adjacent mesosalpinx revealed a small, well-circumscribed nodule (~3 mm) within the mesosalpinx (Figs 1–3). The nodule was composed of bland polygonal cells with distinct borders and clear to finely granular eosinophilic cytoplasm. Nuclei were round to oval, centrally located, and uniform, without pleomorphism, hyperchromasia, necrosis, or increased mitotic activity. The cells were arranged in nested/trabecular architecture resembling zona fasciculata; no medullary component was identified. These features supported the diagnosis of ectopic adrenal cortical tissue.

The postoperative course was uncomplicated and the patient was discharged on postoperative day 3. No additional evaluation or treatment was required because the finding was incidental and there were no clinical features of adrenal hormone excess.

## Discussion

Adrenal development involves two distinct lineages: the cortex arises from mesodermal cells in the urogenital ridge, whereas the medulla derives from neural crest cells that migrate into the cortical primordium. Displacement of cortical tissue during embryogenesis can lead to ectopic adrenal rests, most often along the path of gonadal descent, consistent with the shared embryologic origin of adrenal cortex and gonadal structures [6–8]. Accordingly, ectopic adrenal tissue is typically reported near the kidneys, retroperitoneum, spermatic cord, or ovary, and is most frequently encountered incidentally during pediatric groin surgery; reports in adult women are uncommon [4, 5, 9].

Ectopic adrenal rests are usually small (1–5 mm), well circumscribed, and yellow to yellow–brown because of lipid-rich cortical cells. Although usually clinically silent, they are important because they may be mistaken for metastatic or primary neoplasms and, rarely, may undergo hyperplasia or neoplastic transformation [5, 10]. In the adnexal region, the differential diagnosis includes metastatic clear cell renal cell carcinoma, Sertoli-Leydig cell tumor, ovarian hilus cell rests, Walthard cell nests, paraganglioma, and xanthomatous inflammation. Careful attention to architecture and cytology is essential, and immunohistochemistry can be helpful when morphology alone is insufficient. Adrenal cortical rests typically express steroidogenic factor-1 (SF-1), Melan-A, inhibin, and calretinin; synaptophysin may also be positive, whereas epithelial markers are generally negative [7, 9].

In the present case, the lesion was microscopic, well circumscribed, and composed of bland cortical-type cells without infiltrative growth, supporting a benign ectopic adrenal rest. Awareness of this entity and meticulous sampling of adnexal tissues can prevent overdiagnosis and unnecessary additional work-up.
